# Omega-3 Polyunsaturated Fatty Acid Supplementation in Children With Attention-Deficit Hyperactivity Disorder (ADHD)

**DOI:** 10.7759/cureus.93175

**Published:** 2025-09-25

**Authors:** Moein Hassanzadeh Mobini, Andrew J Boileau

**Affiliations:** 1 Department of Surgery, Saba University School of Medicine, The Bottom, BES; 2 Department of Neuroscience and Neurology, Saba University School of Medicine, The Bottom, BES

**Keywords:** adhd, adolescents, attention deficit hyperactivity disorder (adhd), behavioral, children, clinical symptoms, cognition, omega-3 polyunsaturated fatty acids supplementation

## Abstract

Omega-3 polyunsaturated fatty acids (PUFAs) have gained attention as a potential adjunctive treatment for attention-deficit hyperactivity disorder (ADHD) due to their roles in cognitive and neurobiological functions such as attention and focus, impulse control, executive function, working memory and neurotransmitter regulation, as well as brain development, mainly prefrontal cortex. ADHD, characterized by cognitive and behavioral challenges, has limited therapeutic options, which has prompted interest in alternative treatments. This narrative review evaluates six primary studies on omega-3 PUFA supplementation in children with ADHD, identified through a search of the PubMed database using outlined MeSH and text-word terms, and filtered for randomized controlled trials. The outcomes varied across studies, with some reporting significant improvements in cognition and behavior, while others found no omega-3 superiority over placebo. One study identified a potential association between increased erythrocyte omega-3 PUFA levels and improved literacy and attention, while others found that eicosapentaenoic acid and DHA supplementation significantly improved working memory function among children diagnosed with ADHD. Collectively, the studies suggest omega-3 PUFA supplementation as a potentially valuable adjunctive treatment for specific ADHD domains. However, methodological variations, inconsistent outcomes, and sample size limitations necessitate caution in drawing definitive conclusions. Further research with standardized protocols, larger samples, and extended intervention durations is imperative to elucidate the impact of omega-3 PUFA supplementation on ADHD symptoms in pediatric populations.

## Introduction and background

Attention-deficit hyperactivity disorder (ADHD), a neurodevelopmental condition impacting individuals across various age groups, manifests through enduring patterns of inattention, hyperactivity, and impulsivity. These characteristics contribute to substantial challenges in achieving academic and social success [1]. ADHD affects about 5%-7% of youth and 3%-7% of adults globally [2]. In comparison, children in the United States show an even higher prevalence of around 9% with 62% of diagnosed children taking prescription medication [3]. This growing health crisis brings substantial economic and societal burden, with a total annual societal cost of $19.4 billion among children and $13.8 billion among adolescents [4]. Studies have uncovered the myth that ADHD is a childhood disorder, as 90% of children diagnosed with ADHD suffer symptoms into their adult years, which has opened an area of research between increased recognition and overdiagnosis of ADHD [5].

The pathophysiology of ADHD is complex and multifactorial, inclusive of genetic, neurobiological, and environmental factors, namely nutrition [6]. Linked to cognitive and functional deficiencies arising from diffuse irregularities in the brain, ADHD is characterized by diminished sizes in regions like the anterior cingulate gyrus and dorsolateral prefrontal cortex, which explains the deficits in goal-oriented behavior among those affected [2]. Furthermore, there is a decrease in activity within their frontostriatal area, as evidenced by functional magnetic resonance imaging (fMRI) measurements [2]. Understanding these pathophysiological mechanisms is crucial to direct pharmacotherapy and other possible complementary treatment options.

Inattention, one of the key manifestations of ADHD, is the difficulty of staying on task, sustaining focus, and staying organized, which is not driven by disobedience or intellect. Hyperactivity, another symptom, is defined as a constant need to move, including when it is inappropriate, such as fidgeting, tapping, or even talking. Impulsivity refers to the tendency of an individual to act without careful deliberation or struggle with maintaining self-discipline. It may involve a craving for immediate rewards and a challenge in delaying gratification [5]. Someone who is impulsive might interrupt others or make significant decisions without considering the potential long-term repercussions [5]. ADHD is diagnosed according to the Diagnostic and Statistical Manual of Mental Disorders (DSM). The DSM-5 Text Revision criteria require the presence of six or more of the nine inattention and/or hyperactivity-impulsivity symptom clusters during six months, with the onset of symptoms starting before the age of seven. The severity of symptoms should be sufficient to cause impairments in school, work, or other important areas of functioning [7]. Different assessment tools have been used for the improvement of ADHD symptoms following treatments, including Conners' Parent/Teacher Rating Scales (CPRS/CTRS), which is a standardized tool designed to assess behavioral concerns in children and adolescents diagnosed with ADHD [8].

Stimulant medications are often used as first-line treatment for ADHD [9]. These include drugs such as methylphenidate (MPH) and amphetamines such as lisdexamfetamine [9]. The exact mechanism of action of these stimulants in treating ADHD is not fully understood, but it is generally believed that they function by increasing the availability of certain neurotransmitters in the brain, specifically dopamine (DA) and norepinephrine (NE), both of which play a crucial role in attention and activity levels [9]. By simultaneously blocking the reuptake and increasing the release of DA and NE from the presynaptic neuron, stimulants can enhance the efficiency of information processing at pyramidal neurons in the prefrontal cortex [9]. This results in the improvement of symptoms of inattention, hyperactivity, and impulsivity seen in ADHD [9].

Up to 30%-60% of children taking stimulant medication may experience some side effects, which emphasizes the need for individualized dosing, close monitoring, and considering nonmedicinal options [8]. The main side effects and risks of stimulants include increased energy, hyperalertness, satiety, poor appetite, weight loss, low stature, obsessive/compulsive behaviors, potential to trigger tics, insomnia, agitation, risk of cardiac death, sometimes seizures, and stigma from family surrounding stimulant medications [8]. They can also result in physical withdrawal symptoms and tolerance in children [8]. These side effects highlight the necessity to explore alternative treatment strategies, particularly those that target the root cause of the disorder rather than just managing its symptoms [8].

As widely understood, a well-rounded diet rich in essential nutrients, such as vitamins, minerals, amino acids, and fatty acids, is beneficial for overall health and wellness, including cognitive functions such as neuronal development [1]. Polyunsaturated fatty acids (PUFAs) are fatty acids with more than one double bond in their carbon chain [1]. The position of the first double bond from the methyl (omega) end creates two different types: Omega-3 PUFA and Omega-6 PUFA. These are essential fatty acids and must be obtained through the diet. Omega-3 PUFA has anti-inflammatory effects and is associated with cardiovascular, cognitive, and neurodevelopmental benefits [10]. Omega-3 (n-3) PUFAs are shown to have beneficial effects on several brain disorders such as dyslexia, dyspraxia, and autism spectrum disorder [11]. On the other hand, Omega-6 PUFAs are also essential and support cell structure and immune function, but excessive intake may promote proinflammatory states [10]. Eicosapentaenoic acid (EPA) and docosahexaenoic acid (DHA) are both long-chain omega-3 PUFAs, with EPA mostly having an anti-inflammatory function and DHA more for brain development and cognition [11].

Early studies done in the late 1980s, on the role of essential PUFA supplementation in children with hyperactivity, utilized omega-6 (n-6) long-chain polyunsaturated fatty acids (LCPUFA) but demonstrated minimal advantages. Subsequently, in the 2000s, the majority of studies incorporated a blend of omega-3 and omega-6 LCPUFA, which emphasized EPA (n-3) supplementation with positive outcomes and linked lower scores on ADHD rating scales (ADHD-RS) with reduced omega-3 levels in comparison to omega-6 [12].

Research on the role of omega-3 in brain development and neuronal phospholipid membrane development is ongoing [1]. Simply, they are involved in the synthesis of lipids, such as EPA and DHA, and active forms of n-3 PUFAs, which are essential components of the neuronal membranes in the brain [11]. These membranes regulate the movement of ions across the cell membrane, crucial for nerve-cell communication [11]. Omega-3 fatty acids have anti-inflammatory properties and the ability to affect neuronal cell membrane fluidity, promoting better neurotransmitter function, neurogenesis, and synaptic plasticity [11]. EPA (anti-inflammatory subtype of Omega 3) competes with arachidonic acid (AA; omega-6 PUFA) for enzymes like cyclooxygenase and lipoxygenase, reducing the synthesis of proinflammatory prostaglandins and leukotrienes [10]. DHA component supports neuronal membrane integrity and brain development [11]. Therefore, a deficiency in omega-3 PUFAs can potentially affect brain development and function [10]. This aligns with the meta-analysis done by Chang et al., reporting that children and adolescents diagnosed with ADHD have lower levels of omega-3 PUFAs compared to their counterparts [13].

A study suggested that omega-3 PUFA supplementation could potentially improve clinical symptoms in children with ADHD, especially in those with a low baseline n-3 index. Higher dose of EPA seems to be beneficial in those with higher inflammation when applied for 16-24 weeks [14]. However, a meta-analysis of 22 studies with 1,789 participants, which combines different preliminary studies, found that omega-3 PUFAs might not significantly improve ADHD symptoms, mainly inattention, hyperactivity, and impulsivity, compared to placebo [15]. Therefore, it seems that the current literature is still unclear because of different factors such as possible “contamination” with omega-6, low-dose EPA, especially in those ADHD patients who are omega-3 deficient, shorter duration of intervention, and other comorbid conditions. Evidently, in the subgroup analyses of the studies with at least four months of intervention, omega-3 PUFAs were found to significantly improve ADHD symptoms, mainly inattention, hyperactivity, and impulsivity, compared to placebo [15]. These findings suggested that long-term supplementation may have potential benefits to be explored. Considering the confounding impact of omega-6 on the outcomes, another randomized, double-blind, placebo-controlled trial, using a specific omega-3/6 dietary supplement for six months, conducted on children with moderate ADHD symptoms, found no significant superiority of omega-3/6 supplementation over placebo in ameliorating inattentive symptoms [16]. Overall, while these studies have shown contradicting outcomes, further research using high-quality methodology, specifically the randomized controlled trials (RCTs) as used in this review, is required to determine the potential benefits of omega-3 supplementation in children with ADHD.

This study proposes that omega-3 PUFA supplementation improves the clinical symptoms of children diagnosed with ADHD. The rationale behind this is based on growing evidence that several mental disorders, including ADHD, are likely products of an interplay between genetic susceptibility and environmental factors, one of which could be inadequate nutrition [17]. The dietary intake of omega-3 PUFAs is crucial for physical and mental health and neurodevelopment. However, many diets, especially in Western societies, provide a larger intake of n-6 PUFAs than n-3 PUFAs, resulting in a potential deficiency of omega-3 PUFAs, as it is proposed that they are metabolized by the same enzymatic system [10]. The harmful effects of stimulants, especially in children, also underscore the importance of alternative treatment options [8]. The clinical trials analyzed in this study, five double-blinded placebo-controlled trials and one randomized control three-way crossover design, aim to explore the potential benefits and efficacy of omega-3 PUFA supplementation in children and adolescents with ADHD.

## Review

Methods

**Figure 1:**
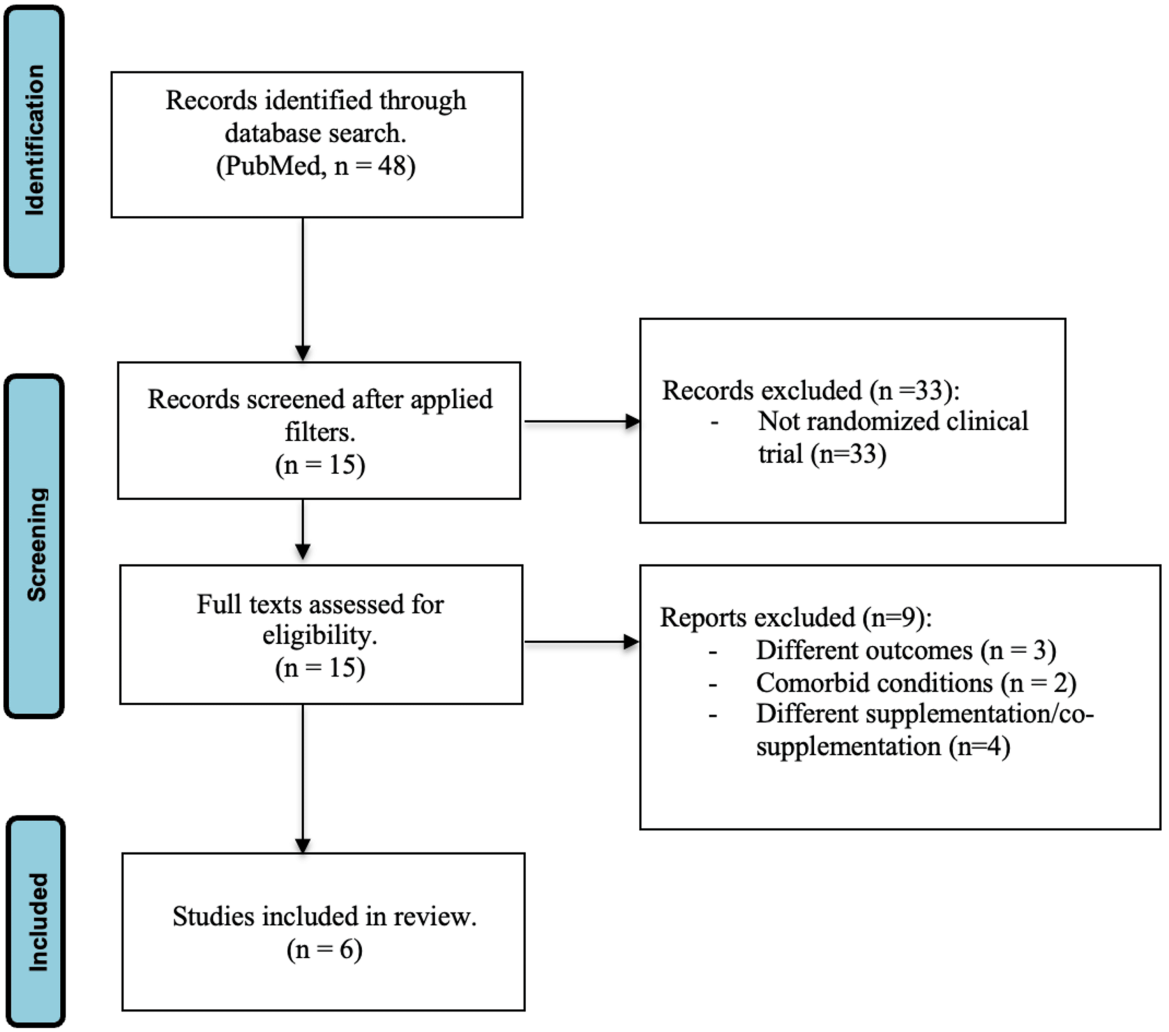
PRISMA-style flowchart for data selection PRISMA: Preferred Reporting Items for Systematic Reviews and Meta-Analyses

The PubMed database was utilized, due to consistency and more relevance, with the following Medical Subject Headings (MeSH): “(Attention Deficit Disorder with Hyperactivity OR ADHD OR Attention Deficit Disorder) AND (Child OR Adolescent) AND [Omega 3 fatty acids OR (DHA OR Docosahexaenoic acid) AND (EPA OR Eicosapentaenoic acid)] AND (Clinical symptoms OR symptoms)” yielding 48 total publications with no time restriction and written in English. The inclusion criteria were confined to RCTs, exploring the impact of omega-3 fatty acids on children and adolescents aged 6-18 diagnosed with ADHD. Within the screening phase, 33 studies were excluded because they were not randomized controlled studies. Exclusion criteria encompassed clinical trials, meta-analyses, systematic and other reviews, studies involving adults (age >18), those addressing coexisting psychiatric conditions (e.g., autism spectrum disorder or ASD, Tourette’s disorder), and those examining differing outcomes (such as omega-6 supplementation, or cosupplementation with zinc or Mediterranean diet). Comorbid conditions such as ASD were excluded due to the complexity of different chronic conditions and their impact on the body's function, as well as different prescribing medications, which might impact the outcome. After assessing full texts for eligibility, another nine studies were excluded due to using omega-6 supplementation, involving comorbid conditions, and measuring different outcomes per the set exclusion criteria. Out of the 48 initial PubMed results, six were identified as primary sources for data analysis (see Figure 1) [18]. The included studies were published between 2010 and 2022. The criteria used to determine the level of evidence included study design, where systematic reviews and meta-analyses of randomized clinical trials represent the highest level (Level I) and expert opinion represents the lowest (Level V), as well as risk of bias, consistency, precision, directness, and magnitude of effect [19]. Figure 1 shows the PRISMA-style flowchart for data selection [18].

Results

Five peer-reviewed double-blinded placebo-controlled randomized clinical trials and one randomized controlled three-way crossover design [20] are included in this analysis, all of which study the outlined population of children and adolescents (aged 6-18 years) diagnosed with ADHD by DSM criteria. Considering the six papers included in the review, the sample size was small, which was considered one of the main limitations of this review. The main explanation for this is the strict inclusion criteria. The main overlapping outcomes studied are ADHD symptom rating scales, cognitive and behavioral assessments, and physiological measures of fatty acid profiles to compare baseline and postintervention EPA/DHA levels in relation to their specific studied outcomes. A summary of all studies is provided in Table 1, with additional comparative details presented in Table 2. All included studies were considered level I of evidence, which represented the highest quality and most reliable evidence in evidence-based practice and included systematic reviews or meta-analyses of RCTs and high-quality, well-conducted RCTs with low risk of bias, adequate blinding, and robust statistical methods [19].

**Table 1:** Evidence table DSM-IV: Diagnostic and Statistical Manual of Mental Disorders IV; ADHD: attention-deficit hyperactivity disorder; EPA: eicosapentaenoic acid; DHA: docosahexaenoic acid; CBCL: Child Behavior Checklist; LC-PUFA: long chain polyunsaturated fatty acid; HVA: homovanillic acid; fMRI: functional magnetic resonance imaging; DSM-5: Diagnostic and Statistical Manual of Mental Disorders V; CPT: Continuous Performance Test; CI: confidence interval; CPRS-R: Revised Conners' Parent Rating Scale; CDI: Children’s Depression Inventory; WIAT-III: Wechsler Individual Achievement Test-Third Edition; RBC: red blood cell; CTRS: Conners' Teacher Rating Scale; ES: effect size; RG: reference group; KiTAP: Test Battery for Attentional Performance for Children; TEA-Ch: Test of Everyday Attention for Children; DISYPS-II: Diagnostic System for Mental Disorders in Children and Adolescents-Second Edition; ADD: attention-deficit disorder

Study	Date of publication	Study design	Level of evidence	Study population	Therapy or exposure	Outcome/results
Bos et al. [21]	March 19, 2015	16-week intervention, double-blind, randomized, placebo-controlled design	Level 1	40 boys between 8 and 14 years of age with a DSM-IV diagnosis of ADHD	Four groups: children with ADHD receiving either placebo or omega-3 fortified margarine, 10 g of margarine daily, enriched with either 650 mg of (EPA)/(DHA) each or placebo (ADHDPlacebo and ADHDActive, respectively), and children from the reference group receiving the same treatment (RGPlacebo and RGActive)	Primary outcome: reduction in CBCL attention problems in the ADHD group with omega-3 (p < 0.001). No significant reduction in the reference group. Secondary outcomes: higher %DHA in phospholipids in the supplemented group at follow-up (p = 0.001). Negative correlation between CBCL attention problems and omega-3 LC-PUFA status. No significant effects on urinary HVA-to-creatinine ratio. No impact on fMRI results
Chang et al. [14]	November 20, 2019	12-week, double-blind, placebo-controlled trial	Level 1	6-18 years, with the (DSM-5) diagnosis of ADHD with either inattention (attention-deficit disorder, ADD), hyperactivity, or combined presentation	One hundred and three youth were recruited and randomized to n-3 PUFAs (1.2 g/day EPA) or placebo (1.2 g/day soybean oil) for 12 weeks, and 92 subjects (mean age 9.49 + 3.05 years, 85.9% male) completed the 12-week trial	Primary outcome: focused attention (CPT). The EPA group showed greater enhancement (ES = 0.38, CI = -0.05 to 0.80, p = 0.041). Stratification analysis based on endogenous EPA levels: low baseline EPA associated with more pronounced improvements in attention-related measures. The highest endogenous EPA group improved less in impulsivity compared to placebo (ES = -0.83, CI = -1.59 to -0.02, p = 0.022). Changes in EPA levels, total n-3 PUFAs, and n-6/n-3 ratio were significant in the EPA group. No significant differences in biological markers
Cornu et al. [22]	October 5, 2018	Randomized 1:1 placebo-controlled trial 12 weeks	Level 1	Children aged 6-15 years with an established diagnosis of ADHD	Receive either supplements containing DHA and EPA or a placebo for 3 months. EPA 336 mg and DHA 84 mg; for children aged 9-11 years, EPA 504 mg and DHA 126 mg, and for children aged 12-15 years, EPA: 672 mg and DHA 168 mg; capsules also contained 100 µg vitamin A, 1.25 µg vitamin D, and 3.5 mg vitamin E	Primary outcome: parent-rated 18-item ADHD-RS-IV. The placebo group showed a significant reduction (-19%), the DHA-EPA group exhibited a smaller reduction (-9.7%), and a significant difference favors placebo (p = 0.039). Secondary outcomes: no significant differences in CPRS-R, CDI scores. Reading age increased similarly. KiTAP tests revealed baseline differences, with longer reaction times in the placebo group (p = 0.02). Compliance is good (97.5%). Adverse events are reported in both groups. Two severe adverse events in the DHA-EPA group led to hospitalization
Milte et al. [20]	November 8, 2013	16 weeks in a crossover design	Level 1	90 children with ADHD	Children were randomized to consume supplements high in EPA, DHA, or linoleic acid (control) for 4 months each	Primary outcomes: no significant treatment effects on literacy (WIAT-III) and cognition (TEA-ch, Go/No-go task). Increases in erythrocyte EPA, DHA, and total n-3 PUFA associated with improvements in word reading, spelling, attention, and parent-rated behavior (p < 0.05). Negative associations between n-6 PUFA and outcomes. No significant differences in adverse events
Gustafsson et al. [23]	June 8, 2010	Randomized, double-blind, placebo-controlled 15-week trial	Level 1	128 children aged 7-12 years with ADHD	EPA supplementation (PlusEPA: 500 mg EPA, 2.7 mg DHA, 10 mg Vitamin E) or placebo (less than 10% n-3 in PlusEPA for taste, rape seed oil, and medium-chain triglycerides)	Successful 159% rise in EPA concentration in serum and 160% rise in RBC membranes in the PlusEPA group. No significant differences in primary efficacy variables (CPRS/CTRS) overall. Subgroup with oppositional behavior showed significant improvement in CPRS + CTRS (ES 0.51, p = 0.026). Improvement in CTRS scores in the PlusEPA-treated group (ES 0.63, p = 0.01). Improvement in CTRS scores for children below median in hyperactivity/impulsivity on Qb-test (36% with EPA vs. 18% with placebo, p = 0.177)
Widenhorn-Müller et al. [24]	May 27, 2014	Randomized, double-blind, placebo-controlled 16-week trial using standardized rating scales and questionnaires	Level 1	95 children diagnosed with ADHD according to DSM-IV criteria	720 mg of omega-3 fatty acids (600 mg EPA, 120 mg DHA) and 15 mg of vitamin E as an antioxidant. Placebo treatment consisted of two olive oil-containing capsules per day. Behavior was assessed by parents, teachers, and investigators using standardized rating scales and questionnaires. For a subgroup of 81 participants, erythrocyte membrane fatty acid composition was analyzed before and after the intervention	Primary outcomes: no significant changes in parent-rated ADHD symptoms (DISYPS-II). Decrease in parent-rated thought problems in the placebo group. Significant improvement in working memory function (p < 0.05) in the EPA/DHA group compared to placebo. Positive correlation between increased DHA levels and improved parent-rated social problems (p < 0.05). Significant increases in EPA (378%), DHA (37%), and other omega-3 fatty acids. Decrease in omega-6 fatty acids

**Table 2:** Comparative outcomes ADHD: attention-deficit hyperactivity disorder; CPRS: Conners' Parent Rating Scale; CTRS: Conners' Teacher Rating Scale; EPA: eicosapentaenoic acid; DHA: docosahexaenoic acid; ADHD-RS-IV: Attention-Deficit Hyperactivity Disorder Rating Scale IV; CPT: Continuous Performance Test; SNAP-IV: Swanson, Nolan, and Pelham IV; SDQ: Strength and Difficulties Questionnaire; CBCL: Child Behavior Checklist; HAWIK-IV: Hamburg Wechsler Intelligence Scales for Children-IV; SWAN: Strengths and Weaknesses of ADHD symptoms and Normal Behavior; fMRI: functional magnetic resonance imaging; ES: effect size

Study	Population	Main scale used	Comparative outcome
Gustafsson et al. [23]	128 children (7-12 years) with ADHD	CPRS/CTRS	No overall difference vs. placebo; subgroup (oppositional) improved (ES 0.51, p = 0.026)
Milte et al. [20]	90 children (6-13 years) with ADHD	CPRS + literacy/attention tests	No significant treatment effect; ↑ erythrocyte EPA/DHA linked to improved literacy/attention (p < 0.05)
Cornu et al. [22]	162 children (6-15 years) with ADHD	ADHD-RS-IV (parent-rated)	Placebo > EPA/DHA: ADHD-RS-IV ↓19% vs. ↓9.7% (p = 0.039)
Chang et al. [14]	103 youth (6-18 years) with ADHD	CPT, SNAP-IV, SDQ	EPA improved focused attention (ES 0.38, p = 0.041); stronger in low baseline EPA subgroup
Widenhorn-Müller et al. [24]	95 children (6-12 years) with ADHD	DISYPS-II, CBCL, HAWIK-IV	Working memory improved (p = 0.019); no significant change in core ADHD symptoms
Bos et al. [25]	40 boys (8-14 years) with ADHD + 39 controls	CBCL, SWAN	Attention problems ↓ with omega-3 (p < 0.001); no fMRI changes; benefit in ADHD + controls

Effect of omega-3 supplementation with exclusion of stimulant use

A study by Gustafsson et al. [23] aimed to measure the efficacy of EPA in treating children with ADHD diagnosed using DSM-IV criteria. It was an RCT of EPA or placebo over 15 weeks in children aged 7-12 years with ADHD. The inclusion criteria consisted of age between 7 and 12 years old, clinical diagnosis of ADHD combined type using DSM-IV criteria A-E, no prior treatment with stimulant medication, children with any neuropsychiatric comorbidity, as long as pharmacologic treatment had not yet begun, and receiving informed consent. The study assessed ADHD symptoms in children with respect to the degree of cognitive impairment, hyperactivity, oppositional behavior, and overall symptoms' severity. The children received either one capsule daily of PlusEPA (containing 500 mg of EPA, 2.7 mg of DHA, and 10 mg of Vitamin E mixed tocopherols) or a placebo (containing less than 10% of omega-3 in PlusEPA capsules for taste, and a mixture of rapeseed oil and medium-chain triglycerides). The study recruited 128 children, with 92 children included in the intention-to-treat (ITT) analysis. The study's exclusion criteria included mental retardation, autism, major depression, epilepsy, other neurological or endocrinological disorders, fish allergy, and other ongoing medications such as stimulants, and if a child had taken long-chain PUFA, a wash-out period of 10 weeks was required. The socioeconomic status of the families was similar to that of the general Swedish population, and all children were Caucasian [23].

The study used “CPRS 35/CTRS 35” to assess ADHD symptoms in children with respect to the degree of cognitive impairment, hyperactivity, oppositional behavior, and overall severity of ADHD. Fatty acid profiles were measured using gas chromatography, both in serum and red blood cell membranes. Interestingly, as a measure for hyperactivity, the study employed a “continuous performance computerized test combined with an infrared motion analysis system (Qb test).” Blood samples for the analysis of PUFA and dietary habits were recorded at various time points during the study, including baseline and 15-week follow-up. Compliance with treatment was monitored by pill counts, and the patients and guardians were asked standardized questions about any symptoms or adverse experiences at each follow-up visit [23].

Following intervention, the PlusEPA group had a successful 159% rise in their EPA concentration in serum from 1.17 ± 0.44 (mean ± SD) at baseline to 3.03 ± 0.84 at 15 weeks, and a 160% rise in RBC membranes from 0.92 ± 0.26 at baseline to 2.39 ± 0.45 at 15 weeks. The study found no significant differences between the PlusEPA and placebo groups in the primary efficacy variables, as measured by the CPRS and CTRS. However, in a subgroup of children with oppositional behavior problems at baseline, the primary efficacy variable (CPRS + CTRS) significantly improved following EPA supplementation (effect size, ES = 0.51, p = 0.026). The authors stated that this difference may be attributed to variability seen in the CTRS scores between the two groups, with a mean score of 12 compared to a 1.5 score seen in the placebo group (ES = 0.63, p = 0.01). Children scoring below the median on the Qb-test for hyperactivity showed improvement in CTRS scores with EPA supplementation (36%) compared to placebo (18%) with an ES of 0.41; however, this difference was not statistically significant (p = 0.177) [23].

The ratios between omega-6 and omega-3 PUFA (linolenic acid, LA/alpha-LA omega-3, AA omega-6/EPA, and AA/DHA) decreased significantly from baseline to 15 weeks in the PlusEPA group, while they remained unchanged in the placebo group or even increased [23]. "CPRS/CTRS" was used to determine the groups, and to specify more, it was mentioned in this paragraph that a subgrouping was done to identify in more detail responders (ODD-R) and nonresponders (ODD-NR) to EPA supplementation, and analyzed them to assess the effects of omega-3 in these subgroups. In a group of children exhibiting oppositional behavior based on CTRS ratings, two subgroups were identified as ODD-R and ODD-NR to EPA supplementation. While at the 15-week follow-up, fatty acid profiles did not differ between these two subgroups. At baseline measurements, the ODD-R group showed significantly lower EPA levels, 1.01 ± 0.22 (mean ± SD), compared to the ODD-NR group, 1.55 ± 0.59 (p = 0.025). Moreover, there was a significantly higher total omega-6/omega-3 ratio in serum, 7.00 ± 1.95 in the ODD-R group compared to 5.41 ± 1.63 in the ODD-NR group (p = 0.056) at baseline [23].

Milte et al. [20] designed a randomized controlled three-way crossover trial that aimed to investigate the effects of omega-3 PUFA supplementation on literacy, cognition, and behavior in children with ADHD. The study involved 90 children aged 6-13 years who were deemed eligible to participate if they had a diagnosis of ADHD or parent-rated symptoms >90th percentile on the "CPRS" and "parent-reported learning difficulties." Children were excluded if they had taken omega-3 PUFA supplements three months before the study or were taking ADHD medication [20].

The study involved three interventions: EPA-rich fish oil, DHA-rich fish oil, or safflower oil (control). Subjects ingested four capsules daily, each containing the specified dosage for the respective treatment conditions: EPA-rich fish oil, with 1,109 mg EPA and 108 mg DHA; DHA-rich fish oil, with 264 mg EPA and 1,032 mg DHA; or safflower oil (control), with 1,467 mg linoleic acid (LA, omega-6 PUFA) daily. All oils were fortified with a minimal amount of Vitamin E [20].

Primary outcomes of interest in the study by Milte et al. [20] included literacy, cognition, and behavior. The study found no significant treatment effects for literacy, as measured by both the Wechsler Individual Achievement Test-Third Edition and the Wechsler Intelligence Scale for Children-Third Edition subtests. The first subset was related to academic achievement, such as reading, spelling, math problem solving, etc., and the second subset was related to cognitive ability and IQ. However, within-subject regression analysis showed that increases in erythrocyte EPA, DHA, and total n-3 PUFA were associated with improvements in word reading (r = 0.203, p = 0.019) and spelling scores (r = 0.365, p < 0.001). Regarding cognition, there were no significant treatment effects, as measured by the TEA-ch and computerized Go/No-go task. However, within-subject regression analysis showed that increases in erythrocyte EPA, DHA, and total n-3 PUFA were associated with improvements in attention, as measured by the “Sky Search” (r = -0.540, p < 0.001) and “Creature Counting tasks” (r = 0.189, p = 0.03) [20].

Moreover, the study found no significant treatment effects for parent-reported behavior, as measured by the CPRS-Long Version. However, within-subject regression analysis showed that increases in erythrocyte EPA, DHA, and total n-3 PUFA were associated with improvements in parent-rated behavior, including oppositional behavior (r = -0.301, p < 0.003), hyperactivity (r = -0.310, p < 0.001), and ADHD index (r = -0.198, p = 0.023). This index is a subset score derived from standardized behavior rating scales such as the Conners' Rating Scales-Revised, which were tools used to assess symptoms associated with ADHD in children and adolescents. In contrast, increases in erythrocyte n-6 PUFA and the n-6 ratio were associated with negative outcomes, including increased oppositional behavior (n-6: r = 0.248, p = 0.004, and n-6/n-3: r = 0.233, p = 0.007) and hyperactivity (n-6: r = 0.260, p = 0.003, and n-6/n-3: r = 0.290, p < 0.001). Compliance and safety were assessed through regular follow-ups. Twelve subjects reported minor adverse events over the 12-month period, including bad breath, gastrointestinal symptoms, itchy skin, an unpleasant taste, nosebleeds, skin rashes, and yellow teeth. There were no significant differences in adverse events between the treatment groups [20].

Cornu et al. [22] conducted a randomized, double-blinded, placebo-controlled clinical trial involving 6-15-year-old children referred for hyperactivity symptoms, with the primary objective to assess the impact of omega-3 fatty acids on ADHD symptoms. The study included 162 children diagnosed with ADHD, randomly assigned to receive omega-3 supplements (DHA-EPA) or a placebo for three months. Utilizing the recommended dietary intakes and doses from earlier studies, the intervention involved administering a daily supplement. The dosage for children aged six to eight years was 336 mg of EPA and 84 mg of DHA; for those aged 9-11 years, it included 504 mg of EPA and 126 mg of DHA, and for children aged 12-15 years, it was 672 mg of EPA and 168 mg of DHA. Additionally, each capsule also contained 100 μg of vitamin A, 1.25 μg of vitamin D, and 3.5 mg of vitamin E. The placebo capsules were made of olive oil but included 18% EPA and 12% DHA (a total of 4.83 mg) plus the same amount of trace vitamins for taste and smell purposes. Compliance was assessed by pill count, and strawberry flavor was added to improve compliance. Exclusion criteria for participants in the study included a documented intolerance to omega-3 fatty acids, the use of fatty acid/fish oil dietary supplements for over one week in the three months leading up to inclusion, or the consumption of MPH or other ADHD medications within the month preceding inclusion. Baseline characteristics were similar between groups [22].

The main outcome of the assessment was the “parent-rated 18-item Attention-Deficit Hyperactivity Disorder Rating Scale IV (ADHD-RS-IV)” evaluated at both baseline and three months. Secondary measures encompassed the alterations in scores observed between baseline and the three-month mark, drawn from various scales including the extended version of the revised “Conners Parent Rating Scale Long Version,” the "L'Alouette" reading test in French, the “battery of Attentional Performance Tests for Children (KiTAP for 6-10 years and TAP for 11-15 years),” and the “Children’s Depression Inventory (CDI)” [22].

Following three months of intervention, the placebo group demonstrated a significant reduction in the ADHD-RS-IV score, showing a mean relative change of -19% (95% confidence interval, CI: -26 to -12). The group receiving omega-3 fatty acids (DHA-EPA) exhibited a smaller score reduction -9.7% (95% CI: -16.6 to -2.9), with a significant difference favoring the placebo group (p = 0.039). Sensitivity analysis accounting for the missing ADHD-RS-IV questionnaire data also confirmed this statistically significant difference (p = 0.023). Longitudinal analysis also revealed a smaller score reduction following three months of intervention, in the DHA-EPA group compared to placebo (mean difference of 1.2 points per month, 95% CI: 0.2-2.3 points, p = 0.026). Secondary analyses of CPRS-R and CDI scores failed to reveal noteworthy distinctions between the treatment groups. Both groups exhibited similar improvements in reading age, as assessed by the L'Alouette test. Baseline disparities were evident in KiTAP tests, with the placebo group displaying a higher number of false responses in the Go/No-Go test (4.8 vs. 6.3, p = 0.016; errors not reported) and a prolonged reaction time (mean difference 140 ms, p = 0.02). However, by the three-month follow-up, only the placebo group showed extended reaction times (mean difference 101 ms, p = 0.02). Compliance was good overall (97.5%, n = 153). Safety analysis reported adverse events in both groups, with 14.9% in the DHA-EPA group and 10.7% in the placebo group. Two severe adverse events occurred in the DHA-EPA group, which led to hospitalization due to their ADHD symptom exacerbation [22].

Another double-blinded, placebo-controlled trial conducted by Chang et al. [14] explored the impact of high-dose EPA supplementation on attention and vigilance in children and adolescents diagnosed with ADHD and low endogenous EPA levels. The participants, aged 6-18 years, were selected based on DSM-5 criteria for ADHD, encompassing inattention, hyperactivity, or combined presentation, with or without oppositional defiant disorder (ODD) symptoms [14].

Participants were either individuals who had not previously used medication or those who had been medication-free for the preceding six months. The trial involved randomizing the participants into two groups: one receiving a placebo (soybean oil 1.2 g/day) for 12 weeks and the other receiving omega-3 (EPA 1.2 g/day) for the same duration. The main assessment criteria involved focused attention, impulsivity, sustained attention, and vigilance, evaluated through the "Continuous Performance Test (CPT)" at both baseline and week 12. Additionally, emotional problems were measured using the "Strength and Difficulties Questionnaire," working memory and short-term memory were evaluated through the "Digit Span Subtest of WISC-IV," and ADHD clinical symptoms were assessed using the "Swanson, Nolan, and Pelham IV," with input from parents, teachers, and the youth [14].

Blood samples were collected at baseline and week 12 to assess erythrocyte levels of PUFAs and levels of plasma high-sensitivity C-reactive protein (hs-CRP) and brain-derived neurotrophic factor (BDNF). The study also included a flowchart detailing study recruitment, which outlined enrollment, randomization, trial completion, CPT assessments, and PUFAs levels for each group. The study provided information on ES and 95% confidence intervals for the results, with a significance level set at p < 0.05 [14].

Statistical analyses revealed that EPA treatment led to improvements in focused attention, as measured by the CPT. The EPA group showed greater enhancement in focused attention compared to placebo, with an ES of 0.38 (95% CI = -0.05 to 0.80, p = 0.041). Stratification analyses showed that individuals with low baseline endogenous EPA levels exhibited more pronounced improvements in attention-related measures, including hit reaction time (HRT) and vigilance (HRT interstimulus interval changes), with ESs of 0.89 (95% CI = 0.10-1.63, p = 0.015) and 0.83 (95% CI = 0.05-1.57, p = 0.036), respectively, compared to the placebo group. While in the highest endogenous EPA group, youth improved less in impulsivity compared to their placebo, based on the commission error, ES = -0.83 (95% CI = -1.59 to -0.02, p = 0.022). As a follow-up, changes in EPA levels, total n-3 PUFAs, and the n-6/n-3 ratio were significant in the EPA group compared to the placebo group (p < 0.0001, p = 0.012, p = 0.008, respectively), but changes in DHA levels were not significantly different. EPA percent change was 0.77 ± 1.12 (mean difference ± SD) for the EPA group vs. -0.15 ± 0.62 in the placebo group (p < 0.0001). Total n-3 PUFA percent change was 0.28 ± 2.30 for the EPA group vs. -0.87 ± 2.58 in the placebo group (p < 0.012). n-6/n-3 ratio change was -0.43 ± 1.83 for EPA group versus 0.99 ± 2.73 in the placebo group (p < 0.008). Biological markers, such as hs-CRP and BDNF, did not differ significantly between groups [14].

Widenhorn-Müller et al. [24] conducted a randomized, double-blinded, placebo-controlled design with a 16-week parallel treatment to investigate the effects of omega-3 fatty acid supplementation on children aged 6-12 years diagnosed with ADHD. The study included 95 children, with boys having an average age of 8.86 years and girls having an average age of 9.10 years. Diagnosis distribution showed that 54.7% of the children were categorized as inattentive, 43.2% exhibited the combined inattentive/hyperactive phenotype, and 2.1% presented predominantly hyperactive/impulsive traits. The exclusion criteria included allergies to fish oil, an IQ below 70, the use of stimulant or psychoactive medication, and the consumption of fatty acid supplements within the preceding six months. The dosing regimen consisted of 720 mg of omega-3 fatty acids (600 mg EPA, 120 mg DHA) and 15 mg of vitamin E daily. Compliance was monitored, and data analysis utilized Mann-Whitney U tests, Wilcoxon tests, t-tests, chi-square analyses, and correlation and regression analyses [24].

Primary outcomes included behavioral assessment, cognitive assessment, and erythrocyte fatty acid profile, which were conducted at baseline and 16 weeks postintervention. Behavioral outcomes were evaluated through standardized questionnaires: “FBB ADHS” (German ADHD-RS), “parent-rated and teacher-rated questionnaires (DISYPS-II),” “Child Behavior Checklist (CBCL),” and “Teacher's Report Form (TRF).” At 16-week follow-up, there were no significant changes for attention, hyperactivity, impulsivity, and total scores (DISYPS-II). However, in parent-rated thought problems, there was a significant decrease observed in the placebo group, 53.74 ± 0.92 (mean ± SE), compared to the EPA/DHA group, 55.34 ± 0.92 (p = 0.03). Cognitive functions were assessed using the “Hamburg Wechsler Intelligence Scales for Children-IV” and the KITAP/TAP.” The study found a significant increase in the “Index Score” for working memory function in the EPA/DHA group, 101.78 ± 11.47 (mean ± SD), compared to the placebo, 96.92 ± 9.73 (p = 0.019). Specifically, the “Digit Span” subtest (Pre: 12.46 ± 2.42, Post: 14.11 ± 2.78, p = 0.003) and “Digits Backward task” (Pre: 5.97 ± 1.35, Post: 6.90 ± 1.90, p = 0.031) scores showed significant improvements with omega-3 supplementation. Erythrocyte fatty acid profile analyses revealed significant increases in EPA (378%), DHA (37%), and other omega-3 fatty acids in the EPA/DHA group postintervention compared to placebo (p < 0.05). Correlation analysis between fatty acid composition and other outcomes indicated a positive association between increased DHA levels and improved parent-rated social problems (r = 0.243, p = 0.032). However, no significant associations were observed between changes in fatty acid levels and other behavioral or cognitive measures [24].

Effect of omega-3 supplementation without the exclusion of stimulant use

Bos et al. [21] employed a double-blinded, randomized, placebo-controlled design to explore the effects of dietary omega-3 fatty acid supplementation on boys with and without ADHD, aged 8-14. A total of 40 boys with a confirmed ADHD diagnosis were recruited using the “Diagnostic Interview Schedule for Children-Parent Version.” The study included a “typically developing” reference group of 39 boys to assess treatment specificity. Participants were randomly allocated to one of four groups: children diagnosed with ADHD who received either a placebo (ADHD Placebo) or omega-3-fortified margarine (ADHD Active), and a reference group subjected to the same interventions (RG Placebo and RG Active). Participants consumed 10 g of margarine daily as part of the dietary supplementation enriched with 650 mg of EPA and DHA. The placebo utilized was a margarine resembling the active product in sensory characteristics but lacking EPA and DHA. The placebo and active products were the same in terms of total saturated fatty acids and omega-6 fatty acids content. Compliance was confirmed through the consumption of at least two-thirds of the test product within the 16-week intervention, with no breaks exceeding seven days [21].

Behavioral measures, assessed using the “CBCL and Strengths and Weaknesses of ADHD symptoms and Normal Behavior scale (SWAN),” revealed that children with ADHD scored higher on attention problems (RG placebo: 2.7 ± 2.8 (mean ± SD), RG active: 2.5 ± 3.6, ADHD placebo: 8.9 ± 3.5, ADHD active: 9.1 ± 2.5, p < 0.001), rule-breaking behavior (RG placebo: 1.4 ± 2.2, RG active: 0.7 ± 0.9, ADHD placebo: 3.8 ± 2.3, ADHD active: 3.1 ± 2.8, p < 0.001), and aggressive behavior (RG placebo: 2.2 ± 3.7, RG active: 2.2 ± 2.7, ADHD placebo: 11.0 ± 5.8, ADHD active: 9.7 ± 7.2, p < 0.001), compared to the reference group at baseline. After the 16-week intervention, there was a significant reduction in CBCL attention problems in both ADHD and reference groups receiving omega-3 supplementation compared to their respective placebo groups (RG placebo: 3.4 ± 2.8, RG active: 2.4 ± 2.6, ADHD placebo: 10.5 ± 3.3, ADHD active: 7.7 ± 3.0, p < 0.001). The Essential Fatty Acids Questionnaire (EFAQ) scores were normalized, but there was no significant effect of omega-3 supplementation on EFAQ scores in either group [21].

As secondary outcomes, physiological measures included the analysis of cheek cell samples for phospholipid fatty acid levels. At baseline, there were no differences between the groups. However, at follow-up, omega-3 supplementation was associated with higher %DHA in phospholipids compared to the placebo (RG placebo: 0.48 ± 0.19, RG active: 0.68 ± 0.24, ADHD placebo: 0.54 ± 0.15, ADHD active: 0.67 ± 0.27, p < 0.001). Correlation analyses indicated a negative correlation between CBCL attention problems and omega-3 LC-PUFA status in the ADHD group at both baseline (r = -0.47, p = 0.048) and follow-up (r = -0.48, p = 0.042). Additionally, the urinary HVA to creatinine ratio, a proxy for DA turnover, showed no significant intervention effects. fMRI results from a cognitive control task revealed no impact of dietary omega-3 supplementation on “task performance or brain activation” [21].

Discussion

This narrative review delved into the analysis of primary sources that explore the potential benefits and efficacy of omega-3 PUFA supplementation in children and adolescents diagnosed with ADHD. The focus is on understanding the impact of omega-3 PUFAs on ADHD symptoms, cognitive and behavioral assessments, and physiological measures of fatty acid profiles. The rationale behind this exploration is based on growing evidence that ADHD is a product of an interplay between genetic susceptibility and environmental factors, one of which is inadequate nutrition. Research provides that dietary intake of omega-3 PUFAs is crucial for physical and mental health and neurodevelopment [10]. Western societies are known to provide a larger intake of omega-6 PUFAs than omega-3 PUFAs, resulting in a potential deficiency of omega-3 PUFAs [5]. Omega-3 PUFAs have beneficial effects through their structural function, maintaining membrane fluidity and neuroplasticity, anti-inflammatory effects, improving mitochondrial function, and reducing oxidative stress [11]. On the other hand, omega-6 PUFA are also essential and support cell structure and immune function, but excessive intake through the Western diet may promote proinflammatory states, which negatively affect brain functions [10]. Additionally, the harmful effects of stimulants, especially in children, including appetite suppression, sleep disturbance, and growth delay, underscore the importance of considering alternative treatment options [8].

There were some major limitations for this review; all included studies applied various scales or questionnaires like the CPRS/CTRS, completed by parents or teachers as the main outcome assessment, which were merely subjective measures of the clinical symptoms of ADHD in their eyes, rather than objective outcomes such as the fMRI or infrared motion analysis system (Qb test) applied as secondary outcomes. Second, some studies either did not measure baseline vs. follow-up assessments of fatty acid profiles or did not stratify participants based on their baseline fatty acid profiles, thereby making it difficult to conclude whether omega-3 supplementation might have any benefits in children with omega-3 deficiency. Another common limitation was poor compliance and high drop-out rates in some studies, resulting in missing data that affected their results and power, leading to misleading reflections. The lack of gender stratification for studied outcomes limits the ability to discern whether observed effects are uniform across genders or if there are subtle variations [25]. In addition, many studies did not adequately control for potential confounding factors such as hormonal changes during adolescence, baseline nutritional status, coexisting psychiatric symptoms, or current dietary and lifestyle patterns, which may significantly influence treatment response. Notably, one of the six studies, Bos et al. [21], exclusively enrolled boys to minimize gender-related confounding on brain activity, while also reflecting the higher prevalence of ADHD in men.

Findings of the Gustafsson et al. [23] Study

Their findings showed that 15 weeks of EPA supplementation improved outcomes in two ADHD subgroups: oppositional and less hyperactive/impulsive children. The study also found that an increase in EPA and a decrease in omega-6 fatty acid concentrations in serum were associated with improvement of ADHD manifestations. This study suggests that the varied responses to treatment among the children might be attributed to the heterogeneity of ADHD, which is known for its diverse manifestations and responses to treatment. Therefore, it is essential to outline the role of fatty acids, highlighting that both omega-3 and omega-6 LCPUFA are crucial for normal brain development and function. The ratio of omega-6 to omega-3 fatty acids, particularly the AA/DHA ratio, appears to be important for membrane fluidity, which is key for neuronal maturation and metabolic functions, along with central nervous system activities such as attention, alertness, and coordination [10].

Gustafsson et al. [23] further explained that the ratio between omega-6 and omega-3 is important to assess, as they compete for the same desaturase and elongase enzyme systems. Therefore, a deficiency of omega-3 or high levels of omega-6 fatty acid intake would shift this metabolic system in favor of omega-6 fatty acid metabolism and diminish the levels of metabolically active omega-3 PUFAs within the brain [10]. Additionally, an explanation offered for the improvement seen in the oppositional subgroup is that a decrease in omega-3 PUFA levels in neuronal tissue has been suggested to potentially reduce serotonin levels during crucial stages of neurodevelopment. This reduction can trigger a series of events leading to suboptimal development of neurotransmitter systems, thereby restricting the regulation of the limbic system, which is already affected in people with oppositional behaviors [11].

This study contributes valuable evidence regarding the potential efficacy of omega-3 PUFA supplementation in children with ADHD. However, the findings may have been influenced by the selective study population, which consisted of Caucasian children from Sweden with socioeconomic backgrounds representative of the general Swedish population [23]. Such homogeneity limits the generalizability of the results to children of other ethnicities, socioeconomic strata, and geographical regions. In addition, the trial used only EPA supplementation rather than a combination of EPA and DHA, which may further restrict the applicability of its findings [23]. Moreover, the study's recruitment process might have inadvertently favored parents with a predisposition toward alternative treatments, such as dietary supplements, for managing ADHD symptoms. This selection bias could skew the study population toward people who are more likely to report positive outcomes from EPA supplementation, thereby affecting the study's external validity and the ability to generalize its findings to the broader ADHD population [23].

Findings of the Milte et al. [20] Study

The study found no significant treatment effects on literacy, cognition, or parent-reported behavior between the three groups (EPA-rich fish oil, DHA-rich fish oil, and control safflower oil). However, within-subject regression analysis revealed associations between increased erythrocyte levels of omega-3 PUFAs (EPA and DHA) and improved literacy, attention, and parent-rated behavior. Conversely, increased levels of n-6 PUFAs were negatively associated with various outcome variables. The study also highlighted the potential benefits of reducing the ratio of n-6:n-3 PUFAs [20].

These outcomes can be further examined by understanding the role of EPA/DHA on brain function. Traditional diets consist of dark leafy vegetables, nuts, seeds, and oily fish rich in essential fatty acids n-3 PUFA with an ideal n-6:n-3 ratio of 1:1. However, the modern diets adopted by the Western culture are mainly from vegetable oils and processed foods with an estimated n-6:n-3 ratio as high as 15-16:1 [10]. Omega-3 PUFA contributes to a range of functions in the body and brain, exhibiting anti-inflammatory, antithrombotic, and vasodilatory effects; while, in contrast, omega-6 PUFA supports inflammation, thrombosis, and vasoconstriction. Hence, an imbalance tipping toward omega-6 PUFA is prone to result in increased inflammation and reduced blood circulation, impacting not only the peripheral system but also the cerebral domain. This, in turn, may contribute to the underlying pathophysiology of mental disorders like ADHD [10,11].

Despite these findings, the study was influenced by a high drop-out rate (37%) due to the extended duration of the study, the requirement for frequent visits to research centers, or difficulty adhering to the supplementation regimen, missing data (incomplete questionnaires), and the lack of a washout effect following each treatment phase. Moreover, the study assumed that the previous treatment would have been washed out during the subsequent supplementation period, as erythrocyte PUFA levels are reported to return to baseline 16 weeks after supplementation cessation. However, it was observed that discontinuing DHA supplementation did not lead to a restoration of DHA levels to their initial baseline. The absence of a washout effect could have influenced the carryover of treatment effects from one phase to the next, potentially confounding the results and contributing to the observed lack of differences between the intervention groups [20].

Findings of the Cornu et al. [22] Study

This study's findings did not demonstrate a significant improvement in ADHD symptoms with omega-3 fatty acid supplementation in children with moderate ADHD. The primary outcome measure, assessing the change in ADHD-RS-IV score over three months, did not reveal a statistically significant difference between the omega-3 and placebo groups. This lack of significant improvement was consistent across various analytical approaches, including per-protocol and ITT analyses. Additionally, the secondary outcome measures related to safety, lexical level, attention, anxiety, and depression did not show significant differences between the two groups [22].

While the study implemented a robust design, the lack of baseline and follow-up assessments for fatty acids, both dietary and serum levels, might explain their discrepant results [22]. Moreover, this study excluded children requiring MPH, which may restrict the generalizability and applicability of the study's results to a more diverse population. However, this exclusion aimed to impede stimulant medication as a confounder, an approach used by previous studies [21]. Additionally, this study underscores the need for further research recruiting children with more varying levels of ADHD to better understand the efficacy of omega-3 in this clinical population. Furthermore, with the results depicting no significant improvement in the intervention group, this may imply the use of omega-3 supplementation as an addition instead of an alternative to the treatment plan for children with ADHD [22].

Findings of the Chang et al. [14] Study

This study found that the EPA group showed a significantly greater improvement in focused attention, as measured by the CPT, compared to the placebo group. After stratification, they found that participants with the lowest baseline endogenous EPA levels from the intervention group demonstrated more improvement than the placebo group in focused attention and vigilance. However, in participants with the highest baseline EPA levels, the intervention group showed less improvement in impulsivity and other ADHD and emotional symptoms compared to the placebo group. As expected, they observed increased blood erythrocyte EPA levels following treatment but no significant effect on DHA levels, hs-CRP, or BDNF plasma levels [14].

These findings suggest that EPA treatment may improve cognitive symptoms in ADHD youth, particularly in those with low baseline endogenous EPA levels, while having minimal impact on those with high baseline EPA levels [14]. This can possibly be depicted as a complex relationship between endogenous/dietary omega-3 PUFA levels and EPA supplementation, specifically on the cognitive symptoms of ADHD. Further, it supports previous research that omega-3 PUFA deficiency is prevalent among children with ADHD [13].

Exploring the main limitations of the study, including patients with comorbidity of ODD, introduces heterogeneity to the recognized population and influences the treatment response. This was justified as youth with ADHD and ODD tend to be less responsive to stimulant treatments. Hence, 51 of 92 participants with ODD were included, and results showed that the improvement in attention was still present in their subgroup analysis [14]. Second, shorter duration of intervention (12 weeks) and not including DHA or other active forms of omega-3 fatty acid in supplementation are considered other limitations to consider [14]. While EPA has been shown to have anti-inflammatory and neuroprotective effects, DHA is also an essential component of brain tissue and has been linked to cognitive function and behavior [11,12,14]. Furthermore, the study did not find any significant effects of EPA treatment on plasma hs-CRP and BDNF levels, which were measured due to the previous supporting evidence of the role of inflammation and neurotrophin factors in the pathophysiology of ADHD, indicating the need for future studies, which would investigate additional biomarkers involved in the effects of omega-3 treatment in ADHD [14].

Overall, Chang et al. [14] endorsed the previously established recommendation for individuals who prefer alternative treatments, such as omega-3 supplements, over stimulants to consider combining at least 750 mg of both DHA and EPA per day for a minimum of 12 weeks. Additionally, they advocate for prioritizing this combined strategy for children displaying signs of low endogenous PUFA levels [14].

Findings of the ​​​​​​​Widenhorn-Müller et al. [24] Study

This study revealed that supplementation with EPA and DHA significantly improved working memory function among children diagnosed with ADHD. Working memory is a cognitive capability essential for temporarily holding and manipulating information, playing a critical role in problem-solving, decision-making, and the learning process. The study also found a significant reduction in parent-rated thought problems in the group that received the omega-3 supplementation. Thought problems refer to difficulties in thinking clearly, concentrating, or making decisions, which cause a significant burden for children with ADHD. These findings suggest a potential cognitive benefit associated with omega-3 fatty acid supplementation in this population. However, the investigation did not detect a substantial impact of the treatment on numerous other behavioral and cognitive aspects, such as attention, hyperactivity, impulsivity, and various dimensions of behavior and cognition. This suggests that the benefits of omega-3 supplementation may be specific to certain cognitive functions and may not extend to all aspects of behavior and cognition in children with ADHD [24].

The authors acknowledged several factors that may have influenced the interpretation of the results. One notable limitation was the relatively small sample size, which could have affected the ability to detect significant effects. Additionally, the recruitment process, which involved primarily obtaining participants through medical professionals and child psychiatrists, may have led to a sample with higher symptom severity, potentially impacting the overall outcomes, as well as affecting the generalizability of the results [24].

Findings of the ​​​​​​​Bos et al. [21] Study

The study investigated the potential benefits of omega-3 PUFA supplementation on symptoms of ADHD in young boys, with a particular focus on inattention. The findings revealed that omega-3 PUFA supplementation may have a positive impact on reducing symptoms of inattention in boys, both with and without ADHD. Notably, the study included a typically developing reference group of boys without ADHD, which allowed for the assessment of the specificity of the treatment to subjects with ADHD, enhancing the study's comprehensiveness and validity [21].

However, the study also encountered several limitations that warrant consideration. Initially, the reliability of detecting DHA in cheek cell samples was compromised for certain subjects due to sample quality. Although the exclusion of their data did not detrimentally impact the statistical analyses, it underscores a practical obstacle in the assessment of PUFA status. The study provides that with the younger population, using minimally invasive techniques instead of drawing blood outweighed the potential for discrepancy in their observed effects. The phospholipid measurements obtained from the cheek samples did not demonstrate the anticipated magnitude of distinction between the placebo and intervention cohorts. Nevertheless, variations persisted between the active product and the placebo within the intervention group, aligning with observed behavioral outcomes. Reflecting that while the differences in the fatty acid profile may not have been substantial, they still demonstrated associations with behavioral outcomes and potentially contributed to the observed effects of the intervention on ADHD symptoms [21].

The low response rate from the TRF limited the amount of data available from an educational context and reduced the strength of the findings related to the children's behavior outside the home. Another limitation pertained to changes in medication for a small number of participants with ADHD during the intervention period. While reanalysis excluding these participants demonstrated that the intervention's effect persisted, the potential influence of medication changes on the outcomes necessitates careful consideration [21].

While the study had hypothesized that omega-3 supplementation would improve cognitive control and alter brain activity, as measured by fMRI, no effects were observed in these areas. This would suggest that while omega-3 fatty acids may have some beneficial effects on attention, they do not appear to influence the neural systems related to cognitive control, considering the dose and the length of intervention. However, the fMRI study encountered challenges related to smaller sample sizes due to subject motion, which could have implications for the generalizability of the findings. Despite these limitations, the study's post hoc power analysis indicated that a substantial sample size would have been necessary to detect a significant effect of the intervention on brain activity, underscoring the importance of adequately powered studies in future research endeavors [21].

Integrative Analysis

The primary sources included for analysis were six RCTs with an average duration of 12-16 weeks, enrolling a total of 657 participants across all studies. A study published in Clinical Psychopharmacology and Neuroscience recommended a combination of EPA and DHA at a dosage of ≥750 mg/day for a duration of 16-24 weeks for youth with ADHD [11]. This was based on RCTs and systematic literature reviews that showed potential improvements in clinical symptoms, especially in those with low baseline omega-3 index [11]. The physiological effects of omega-3 PUFA supplementation may require time to become apparent in the body due to the need for the fatty acids to be metabolized into their active forms, such as EPA, DHA, and other oxylipins [11,12,20]. Therefore, given the varying results outlined in this analysis, future research should examine not only the efficacy of longer supplementation periods (>16 weeks) but also the impact of higher omega-3 dosages, as current evidence suggests greater anti-inflammatory and neurocognitive benefits at elevated intake levels [11-13].

A significant facet contributing to the strength and generalizability of our analysis is the broad age spectrum covered in all studies, spanning from 6 to 18 years. This inclusivity ensures a profound understanding of the potential effects of omega-3 supplementation across various developmental stages within the pediatric and adolescent cohorts. Another intrinsic strength lies in the gender-inclusive methodology adopted by all studies except Bos et al. [21], distributing both boys and girls in their randomization process. While this approach enhances the generalizability and reliability of conclusions to a broader population, it also introduces a potential confounder in their findings, as ADHD may manifest differently in boys and girls. A study on gender differences in coexisting symptoms and executive function measures found that girls with ADHD may exhibit more internalizing symptoms, such as anxiety and depression. In comparison, boys may display more externalizing symptoms, such as conduct problems and hyperactivity [25]. These differences in symptoms may be due to the hormonal variations in the developmental stages of boys and girls, which are limited by up-to-date literature, foreseeing an area for further study.

The sample sizes across studies ranged from 40 to 162 participants, which provided a reasonable basis for statistical analysis. Larger sample sizes enhance the precision of estimates and improve the reliability of observed effects. The use of placebo-controlled designs in five of the six studies, with the exception of Milte et al. [20], further supports internal validity by minimizing confounding. However, this review is limited by its narrative design, as no meta-analytic methods were applied.

In all studies, the supplements were given as capsules, while Bos et al. [21] consisted of 10 g of margarine daily with a combination of EPA and DHA. Omega-3 supplementation doses range from 420 mg per day EPA/DHA [22] to 1,300 mg/day EPA/DHA [21]. From all the double-blind placebo-controlled trials, Bos et al. [21] was the only study using EPA and DHA 650 mg each, 1.3 g of daily omega-3 PUFAs, with a 1:1 EPA to DHA ratio, while all other studies used a 3:1 EPA to DHA ratio with the aforementioned average dosing range. The composition and amount of dietary omega-3 supplements varied across studies and were difficult to compare using the different outcome measurements used across the primary sources. Therefore, this opens an arena of improvement for the current literature to investigate a significant dose-response between omega-3 supplementation and clinical symptoms of ADHD.

The evidence reviewed here underscores the potential role of omega-3 supplementation as a complementary intervention for ADHD, but also highlights the need for more rigorous and standardized research. The heterogeneity in dosing strategies, study populations, and outcome measures across trials makes it difficult to draw definitive conclusions. Future studies should, therefore, examine optimal EPA-to-DHA ratios, dose-response relationships, and longer supplementation durations [11,12,19,22], while also recruiting larger and more diverse populations to improve generalizability. Incorporating standardized assessment tools and considering relevant confounders, such as baseline nutritional status and coexisting psychiatric conditions, will be essential for clarifying the clinical utility of omega-3 fatty acids in ADHD management [11,12].

## Conclusions

This review of six trials on the efficacy of omega-3 supplementation in children with ADHD has identified significant limitations that affect the interpretation of the results. The primary concern is the inconsistency in assessment tools across the different trials, which poses a difficulty when comparing the outcomes between studies leading to mixed findings. Using various scales and questionnaires, each with its own scoring and interpretation poses a challenge for synthesizing data and drawing definitive conclusions. Despite these methodological constraints, the potential of omega-3 supplementation as a complementary treatment for ADHD remains an area of interest, especially for children with deficient baseline levels of omega-3. Some studies suggest that these children may benefit more from supplementation, although further research is needed to confirm this.

The anti-inflammatory effects of omega-3 fatty acids, together with their role in neuronal function and neurotransmitter regulation, may contribute to the management of ADHD symptoms. However, it is essential to note that the current evidence does not support omega-3 supplementation as a complete alternative to traditional ADHD treatments. Instead, it may serve as a complementary approach, potentially enhancing the effectiveness of other treatments and improving overall outcomes for children with ADHD. Future research should strive for standardization in outcome measures and address other noted limitations, such as sample size and participant compliance, while also investigating dose-response effects to establish proper dosing guidelines. These efforts will provide more conclusive evidence on the role of omega-3 fatty acids in ADHD management.
